# Reducing Sugar Production from *Teff* Straw Biomass Using Dilute Sulfuric Acid Hydrolysis: Characterization and Optimization Using Response Surface Methodology

**DOI:** 10.1155/2021/2857764

**Published:** 2021-11-02

**Authors:** Andinet Alemayehu Tesfaw, Belachew Zegale Tizazu

**Affiliations:** Department of Chemical Engineering, Addis Ababa Science and Technology University, Addis Ababa, Ethiopia

## Abstract

The present study evaluated first the characterization of *Teff* straw and then Box–Behnken design (BBD), and response surface methodology was adopted to optimize the parameters (hydrolysis temperature, dilute sulfuric acid concentration, solid to liquid ratio, and hydrolysis time) of dilute sulfuric acid hydrolysis of *Teff* straw in order to get a maximum yield of total reducing sugar (TRS). The chemical analysis of *Teff* straw revealed high amounts of cellulose (41.8 wt%), hemicellulose (38 wt%), and lignin (17 wt%). The morphological analysis using SEM showed that hydrolyzed *Teff* straw with dilute sulfuric acid has more pores and distorted bundles than those of raw *Teff* straw. XRD analysis also indicated that hydrolyzed *Teff* straw has higher crystallinity index and smaller crystallite size than raw *Teff* straw, which might be due to removal of hemicellulose, amorphous cellulose, and lignin components. Under the optimized conditions for dilute sulfuric acid hydrolysis of *Teff* straw (120°C, 4% v/v H_2_SO_4_ concentration, 1 : 20 solid to liquid ratio, and 55 min hydrolysis time), we have found a total reducing sugar yield of 26.65 mg/g. The results of validation experiment under the optimum conditions agreed well with model predictions.

## 1. Introduction

Biomass is by far the largest energy provider contributing a total of 1,150 million tons of oil equivalent which translates into a 79% share of the total energy supply [1]. Because of the depletion of fossil fuels and environmental pollution, researchers have been committed to studying the production of value-added chemicals and biofuels originated from lignocellulosic biomass [1]. Lignocellulosic biomass is an abundant and inexpensive source of fermentable sugars for the production of biofuels and value-added chemicals. *Teff* straw is among the lignocellulosic materials used for the synthesis of biofuels and value-added products [2]. Like other lignocellulosic materials, the main components of *Teff* straw are cellulose, hemicellulose, and lignin. *Teff* straw has not been studied much for its potential applications. Dame [2] reported that in Ethiopia an average of 3.7 million tons of *Teff* cereal has been produced per annum; correspondingly large amount of *Teff* straw has been produced during banging, which was more than 2 million tons of *Teff* straw in every year. It has been primarily disposed of through burning in the field, rather than used as animal feed. The disposed of *Teff* straw could cause environmental pollution. Thus, converting to valuable products such as total reducing sugar for subsequent conversion of value-added chemicals and biofuels is a preeminent option instead of burning them and disposing of them to the environment.

Lignocellulosic biomass typically consists of (1) cellulose (30%–50%), a polymer of *β*-linked D-glucose, which is susceptible to enzymatic hydrolysis and easy to metabolize; (2) hemicellulose (15%–40%), primarily C_5_ and C_6_ sugars; (3) lignin (15%–35%), which is difficult to metabolize and is a randomly cross-linked aromatic polymer of phenyl propane units combined by different linkages (C–O–C ethers and C–C), resisting biochemical conversion and demanding high temperatures to convert; and (4) other components such as extractives and ash. Due to its heterogeneous composition, hemicellulose is of particular interest to researchers; i.e., hemicellulose is branched heteropolymers with a degree of polymerization of around 80 to 200 and is depolymerized into its monomeric units, mainly xylose, and their subsequent conversion into biofuels and value-added products via microbial fermentation [3]. Pretreatment is the crucial stage in the transformation of lignocellulosic biomass to fermentable sugars. It is vital to alter the structure of cellulosic biomass for hydrolysis. This is due to the crystallinity of cellulose, degree of polymerization, moisture content, available surface area, and lignin content [4]. Pretreatment has been recognized as one of the most expensive processing steps in lignocellulosic biomass–to–fermentable sugars conversion. The goals of any pretreatment are (1) the increase of the surface area and porosity, (2) modification of lignin structure and removal of lignin, (3) depolymerization of hemicellulose and removal of hemicellulose, and (4) reduction of the crystallinity of cellulose. An effective pretreatment should (1) avoid the need for reducing the size of biomass particle, (2) preserve the pentose (hemicellulose) fractions, (3) limit the formation of degradation products that inhibit the growth of fermentative microorganism, and (4) minimize energy demands; in addition, the pretreatment agent should have low cost and be capable of recycling inexpensively [5]. Pretreatment with concentrated acids such as hydrochloric acid and sulfuric acid can result in enhancement of enzymatic hydrolysis of lignocellulosic biomass to produce fermentable sugars. Even though they are strong agents for cellulose hydrolysis, concentrated acids are toxic, corrosive, and hazardous and thus require reactors that are resistant to corrosion, which makes the pretreatment process very expensive. In addition, the concentrated acid must be recovered after hydrolysis to make the process economically feasible [6, 7]. Dilute acid hydrolysis has been widely used for pretreatment of lignocellulosic biomass. This is due to high hydrolysis rate, being specific toward hemicellulose, and being more economical than enzymatic, concentrated acid and base pretreatment [8]. The yield of fermentable sugars during acid hydrolysis is affected by various factors such as hydrolysis time, solid to liquid ratio, hydrolysis temperature, and acid concentration. Dilute sulfuric acid hydrolysis is the most common and effective pretreatment process for *Teff straw* [9], but other reagents such as hydrochloric, nitric, and phosphoric acids can also be used. Various research works have been done on optimization of dilute acid hydrolysis of different lignocellulosic biomass using response surface methodology. Limited reports have been obtained on the optimization of dilute acid hydrolysis of *Teff straw* to produce total fermentable sugars using response surface methodology. The conventional method of optimization involves varying one factor at a time and keeping the others constant. It might be useful but does not explain the effects of interaction between the various factors under consideration. Thus, the response surface methodology, which is a collection of mathematical and statistical techniques for empirical modeling, was used for optimization of dilute sulfuric acid hydrolysis of *Teff* straw for total reducing sugar production. The present study aimed to statistically optimize the process parameters (viz., sulfuric acid concentration or load, hydrolysis temperature, hydrolysis time, and solid to liquid ratio) of dilute sulfuric acid hydrolysis of *Teff* straw. Prior to response surface methodology followed by Box–Behnken design (BBD) optimization of dilute sulfuric acid hydrolysis of *Teff* straw, the composition of *Teff* straw was first analyzed. Box–Behnken design (BBD) has been used to design and optimize the process parameters of dilute sulfuric acid hydrolysis conditions and the interaction among these parameters on dilute acid hydrolysis of *Teff* straw to obtain maximum total reducing sugars yield.

## 2. Materials and Methods

### 2.1. Materials and Chemicals

*Teff* straw was collected from Debre Zeyit, Oromia Region, Ethiopia, as the raw material for total reducing sugar production. All chemicals and reagents used throughout this study were of analytical reagent (AR) grade and maximum purity from HiMedia and Ranchem, India. The reagents were obtained from Atomic Educational Materials Supply PLC. Sulfuric acid, hydrochloric acid, and methylene blue indicator (purity 98%, 37%, and 100%, respectively) were obtained from Blulux Laboratories Pvt. Ltd. In addition, potassium hydroxide (98.08% pure, HiMedia Laboratories Pvt. Ltd.) was required to determine the total reducing sugar by Fehling solution method. Sodium hydroxide (98.08% pure, HiMedia Laboratories Pvt. Ltd.) was employed for determination of hemicellulose content of *Teff* straw, whereas sulfuric acid, H_2_SO_4_ (LOBA Chemie, purity 98%), was used to determine the lignin content.

### 2.2. Collection and Preparation of *Teff* Straw

*Teff* straw collected from Debre Zeyit, Oromia Region, Ethiopia, was washed three times in order to remove soluble components and dust particles. It was then sun-dried for two days for the next size reduction process. The dried *Teff* straw was ground and screened using a standard sieve for the average particle size of 710 *μ*m (0.71 mm). The particle size was taken based on the previous literature reported elsewhere. The sieved *Teff* straw was stored in a zipped lock plastic bag at ambient temperature until use.

### 2.3. Proximate Analysis of *Teff* Straw

The proximate analysis of *Teff* straw, viz., moisture content, total ash content, total volatile matter, and fixed carbon, was determined using standard procedures of gravimetric methods.

#### 2.3.1. Determination of Moisture Content

This was done by the gravimetric method. About 15 gram of finely powdered *Teff* straw was weighed into the silica crucible; then the crucible was placed without lid in electric hot air oven by maintaining a temperature of 105°C for an hour to evaporate water; and the crucible was then taken out, cooled in desiccator, and weighed for loss in weight. Therefore, weight of *Teff* straw sample before and after heating = (weight of Teff straw sample + weight of crucible) − weight of crucible [10].

(1) $$\begin{array}{c} \text{MC}\left(\%\right)=\left(\frac{g-x}{g}\right)\times 100, \end{array}$$

where *g* is the weight of sample, *x* is the weight of dry matter, and (*g* − *x*) is the loss in weight.

#### 2.3.2. Determination of Ash Content

This was done by the furnace incineration gravimetric method. 3.0 g of the processed *Teff* straw was measured into a previously weighed porcelain crucible. Finley, powdered *Teff* straw in the crucible was heated without lid in a muffle furnace at a temperature of 700°C for 20 minute. Afterward, the crucible was taken out, cooled first in open air, then transferred to desiccator, and weighed. Accordingly, weight of crucible + weight of *Teff* straw before heating was given as weight of crucible + weight of *Teff* straw after heating. Finally, weight of ash = (weight of sample + weight of crucible) − weight of crucible.

(2) $$\begin{array}{c} \text{Ash}\left(\%\right)=\frac{w_{1}-w_{2}}{w_{1}}\times 100, \end{array}$$

where W_1_ is the initial weight of sample before burning and W_2_ is the final weight of sample after burning.

#### 2.3.3. Total Volatile Matter

Total volatile matter is the weight loss obtained on heating. The *Teff* straw in the crucible was covered with a lid, placed in muffle furnace, and maintained at a temperature of 900°C for 9 minutes to avoid toxic matters. After 9 minutes, the crucible was cooled in air; afterward, it was kept into desiccator and weighed. Therefore, weight of *Teff* straw before heating could be total weight − weight of crucible; at the same time, weight of *Teff* straw after heating = (weight of sample + weight of crucible) weight of crucible. Finally, total weight loss of moisture *Teff* straw = weight loss due to volatile matter + moisture. Weight loss due to volatile matter = total weight loss − moisture.

(3) $$\begin{array}{c} \text{VM}\left(\%\right)=\left(\frac{\text{weight loss due to volatile matter}}{\text{weight of }Teff\text{ straw}}\right)\times 100. \end{array}$$

#### 2.3.4. Fixed Carbon

The FC of *Teff* straw can be determined as in the following equation:

(4) $$\begin{array}{c} \text{FC}\left(\%\right)=100-\left(\text{VM}+\text{Ash}+\text{MC}\right)\%, \end{array}$$

where VM is the volatile matter and MC is the moisture content.

### 2.4. Chemical Composition Analysis of the *Teff* Straw

#### 2.4.1. Determination of Extractives

The amount of extractives in *Teff* straw was estimated by using Soxhlet extraction apparatus through extraction thimbles. Acetone 400 mL for 3 g of oven-dried *Teff* straw was used as the solvent for extraction, and the temperature was held at 70°C for a 4 h run period on the heating mantle. The sample was air-dried for few minutes at room temperature. It was then dried at 105°C in an oven until a constant weight was obtained and then cooled in a desiccator [11, 12]. The percent by weight of the extractives content was evaluated as the difference in weight between the raw extractive-laden *Teff* straw and extractive-free *Teff* straw.

#### 2.4.2. Determination of Hemicellulose

2 g of extracted dried *Teff straw* was transferred to a 250 mL Erlenmeyer flask. 150 mL of 0.5 M sodium hydroxide was added. The mixture was boiled for 3.5 h with distilled water so as to increase the heating effect and minimize lime scales that can come from tap water. It was filtered after cooling through vacuum filtration and washed until neutral pH. The residue was dried to a constant weight at 105°C in an oven. The difference between the sample weight before and after this treatment was the hemicellulose content (wt%) of oven-dried *Teff* straw [13, 14].

#### 2.4.3. Determination of Lignin

3.05 g of dried extracted *Teff* straw was weighed in glass test tubes, and 30 mL of 72 wt (%) H_2_SO_4_ was added. The sample was kept at room temperature for 2 h with careful shaking at 30 min intervals to allow for complete hydrolysis. After the initial hydrolysis, 84 mL of distilled water was added in order to get 4 wt (%) H_2_SO_4_ solutions. The second step of hydrolysis was made in an autoclave for 1 h at 121°C. The mixture was then cooled at room temperature. *Teff* straw hydrolysate was filtered through vacuum filtration. The acid insoluble lignin was determined by drying the residues at 105°C and cooled in a desiccator. The acid soluble lignin fraction was determined by measuring the absorbance of the acid hydrolyzed samples at 278 nm. The lignin content (wt%) was determined as the summation of acid insoluble lignin and acid soluble lignin [14].

#### 2.4.4. Determination of Cellulose

The cellulose content (%w/w) was determined by difference, assuming that extractives, hemicellulose, lignin, and cellulose are the only components of the entire biomass.

### 2.5. Acid Hydrolysis for the Production of Total Reducing Sugar

#### 2.5.1. Estimation of Total Reducing Sugar by Fehling Methods

Determination of total reducing sugars was based on the procedures of Lane and Eynon's method. To determine the total reducing sugars, two separate Fehling solutions were prepared and labeled as Fehling's A and Fehling's B. The two solutions were mixed in equal volumes to get the final Fehling's solution, which has a deep blue color [15]. Determination of Fehling factor was the first step for this method, in which 5 ml of each solution, Fehling's A and B, was taken out. 17.32 g of copper sulfate was dissolved in 250 ml of distilled water in the volumetric flask to prepare Fehling A, whereas Fehling B was prepared by using 86.5 g of Rochelle salt (potassium sodium tartrate) and 25 g of sodium hydroxide dissolved in 250 ml of distilled water in the volumetric flask. A 1 g of methyl blue indicator and 1 g of dye phenolphthalein indicator were mixed with distilled water to make up a volume of up to 100 ml and dissolved in ethanol to make up a volume of 100 ml for Fehling factor determination [15].

#### 2.5.2. Standardization of the Fehling's Solution for Invert Sugars

For standardization of the Fehling's solution for invert sugars, 4.75 g of analytical grade sucrose was dissolved with 50 ml distilled water in 500 ml volumetric flask, and at a time 5 ml concentration HCl was added and allowed to stand for 24 hrs. Subsequently, this solution was neutralized with sodium hydroxide using phenolphthalein as endpoint indicator. After uniform mixing, 25 ml of the solution was transferred to a 100 ml volumetric flask, making up to volume (i.e., 1 ml = 2.5 mg of invert sugar) by transferring to the burette having an off-set tip, and titrated against Fehling's solution as described in the following equation:

(5) $$\begin{array}{c} \text{Factor for Fehling's solution}\left(g\text{ of invert suger}\right)=0.0025\times V_{1}\left(\text{ml}\right). \end{array}$$

*Preliminary titration*: 5 ml of each solution, Fehling A and B, was pipetted into 250 ml conical flask. The solution was then mixed uniformly and added to 10 ml water and few pumice stone or glass beads. The sugar solution was dispensed from the burette and then heated to boiling. Three drops of methylene blue indicator were added dropwise to the sugar solution until the blue color disappears to a brick red endpoint.

*Final titration*: 5 ml of each solution, Fehling A and B, was pipetted into a 250 ml conical flask. 0.05 to 1.0 ml sample solution from titer value of the preliminary titration was added to the flask and heated to boiling. Subsequently, 3 drops of methylene blue indicator were added. The titration was completed within 1 min by adding 2 to 3 drops of sugar solution at a time until the indicator was decolorized. Finally, we noted down the titer value when the boiling liquid was changed to brick red color. The volume of clarified sample solution required for Fehling's reaction (titer) was represented as *V*_3_ (ml). Based on the factor for Fehling's solution, *V*_3_ (ml) sample solution contained 0.0025 *V*_3_ g reducing sugar (as invert sugar). To determine the total reducing sugars of *Teff* straw, 50 ml of the hydrolysate from *Teff* straw was pipetted into a 100 ml volumetric flask. Then, 5 ml of concentrated hydrochloric acid was added and allowed to stand at room temperature for 24 hrs, and then the solution was neutralized with concentrated sodium hydroxide solution of 0.1 N using phenolphthalein as endpoint indicator. The titration was performed against Fehling's solution, the total reducing sugars were determined as invert sugars, and the solution was transferred to 50 ml burette having an off-set tip which was represented as titer = *V*_4_ (ml). Based on the factor for Fehling's solution, total reducing sugars in *V*_4_ (ml) = 0.0025 × V_1_ g. 50 ml of the clarified and deleaded solution was diluted twice (50 ml to 100 ml) after hydrolysis. Dilution volume of the hydrolyzed *Teff* straw would be 2 × V_2_ according to the procedure of [16]. The total reducing sugar (as invert sugar %) was determined as in the following equation:

(6) $$\begin{array}{c} \text{total reducing sugar}\left(\text{as invert sugar}\%\right)=\frac{0.5\times V_{1}\times V_{2}}{V_{4}\times W}, \end{array}$$

where 0.5 × *V*_1_ is the Fehling factor (g),*V*_2_ is the volume required for hydrolysis (ml), *V*_4_ is the titer (ml), and W is the weight of the *Teff* straw used in hydrolysis (g).

### 2.6. Scanning Electron Microscope (SEM) Analysis

The modification of surface structure of *Teff* straw was analyzed by SEM [16–20]. SEM analysis has been employed most dominantly in order to determine the microstructure of both raw and acid hydrolyzed *Teff* straw.

### 2.7. X-Ray Diffraction (XRD) Analysis

The effect of the crystal size on the X-ray patterns has been studied in detail by using the origin software. This software considers the peak shape analysis to provide information on crystal size and percentage of crystallinity of samples; on the other hand, a powder diffraction file is required for the analysis of the effect of the crystal size on the X-ray diffraction pattern. In order to analyze the crystalline structure of this *Teff* straw, X-ray diffractometer equipped with copper (Cu) radiant source, energy of 40 kV, electric current of 30 mA, scanning speed of 3^o^/min, and scanning range of 10 to 80 degrees was used. Crystallinity (peak to noise ratio) can be determined using the following equation:

(7) $$\begin{array}{c} \text{crystallinity }=\;\frac{\text{area of crystalline peaks}}{\text{area of all peaks}\left(\text{crystalline}+\text{amorphous}\right)}\times 100. \end{array}$$

From XRD data, area of crystalline peaks and area of all peaks of untreated and treated *Teff* straw, respectively, could be found using OriginPro and Excel software. In XRD data, the broadening (*β*_*T*_) of peaks is due to the combined effect of crystallites size (*β*_*D*_) and macrostrain (*β*_*ε*_) that can be expressed as the following equation:

(8) $$\begin{array}{c} \beta_{T}=\beta_{D}+\beta_{\varepsilon }, \end{array}$$

where *β*_*T*_ is the total broadening, *β*_*D*_ is broadening due to the crystallite size, and *β*_*ε*_ is the broadening due to strain. From the Scherrer equation, the crystallite size was adopted in the equation below.

(9) $$\begin{array}{c} D=\frac{K\lambda }{\beta_{D}\cos\theta }, \end{array}$$

where *β*_*D*_ is the FWHM (broadening of the peak) in radians, K is the shape factor (0.94), *λ* is the wavelength of X-ray source (0.15406 nm), and *θ* is the position of the peak in radians. Similarly, the XRD peak broadening due to microstrain is given by the following equation:

(10) $$\begin{array}{c} \beta_{\varepsilon }=4\varepsilon \;\tan\theta , \end{array}$$

where *β*_*ε*_ is broadening due to strain and *ε* is strain. Substituting (9) and (10) into (8), we get the following equation:

(11) $$\begin{array}{c} \beta_{T}\cos\theta =\varepsilon \left(4\;\sin\theta \right)+\frac{K\lambda }{D}. \end{array}$$

Equation (11) represents a straight line, in which *ε* is the ratio between two lengths (slope) of the line (dimensionless quantity) and *Kλ*/*D* is the *y*-intercept by calculating FWHM and peak position from XRD data. From the data of W-H plot, *y*-intercept is (*Kλ*/*D*) and slope (*ε*) is strain.

### 2.8. Experimental Design

Four-variable (temperature, concentration of H_2_SO_4_, solid to liquid ratio, and hydrolysis time) Box–Behnken design (BBD) with three replicates at the center point was performed to optimize the parameters of dilute sulfuric acid hydrolysis conditions for maximum total reducing sugar (TRS) yield from *Teff* straw. The experimental design consisted of 27 runs including three center points. The experimental data for total reducing sugar (TRS) yield from *Teff* straw was fitted using a second order polynomial function model:

(12) $$\begin{array}{c} Y=\beta_{o}+\sum \beta_{i}X_{i}+\sum \beta_{ii}X_{i}^{2}+\sum \beta_{ij}X_{ij}, \end{array}$$

where *Y* is the response, *β*_*o*_ a constant, *β*_*i*_ the linear coefficients, *β*_*ii*_ the squared coefficients, and *β*_*ij*_ the interaction coefficients.

Coded and actual value levels of the variables for Box–Behnken design are shown in Table 1. The design and levels of variables with experimental total reducing sugar (TRS) yield from the dilute sulfuric acid hydrolysis of *Teff* straw are given in Table 2. Design-Expert statistical software 7 (trial version) was used for the analysis and optimization of the experimental data. Analysis of variance (ANOVA) was performed in order to evaluate the statistical significance of the model.

## 3. Results and Discussion

### 3.1. Proximate Analysis

Table 3 depicts the proximate analysis of *Teff* straw as compared to other biomass sources. The proximate values determined in the present study were moisture content = 6.7%, volatile matter content = 78.5%, fixed carbon = 2.3%, and ash content = 12.5%. As shown in Table 3, the values of proximate analysis determined in this study are comparable to the previous studies.

### 3.2. Determination of Chemical Composition of *Teff* Straw

The chemical composition of *Teff* straw (viz., cellulose, hemicellulose, lignin, and extractive contents) was determined using standard gravimetric procedure.  Table 4 shows the chemical composition of *Teff* straw as compared to other various biomass sources. The compositional analysis of *Teff* straw for the present study was as follows: cellulose = 41.8 wt%, hemicellulose = 38.0 wt%, lignin = 17.0 wt%, and extractive content = 3.2 wt% on dry basis. The results of this study are comparable to other biomass sources. The chemical compositional analysis results revealed that *Teff* straw biomass shows promising sources for biofuels and value-added chemicals production due high contents of cellulose, hemicellulose, and lignin. *Teff* straw having a cellulose content of 41.8% and hemicellulose content of 38% indicates promising source of total reducing sugar.*Teff* straw has almost comparable amount of lignin content to that of other feedstocks; in particular, it has almost the same amount of lignin as wheat straw but higher cellulose which could be sustained at higher temperature during hydrolysis.

### 3.3. SEM Analysis of Raw and Acid Hydrolyzed *Teff* Straw

**Figure 1:**
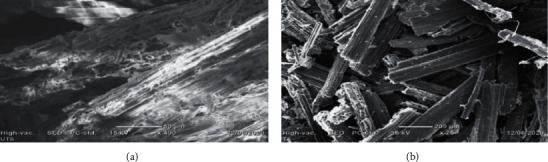
SEM analysis of raw (a) and acid hydrolyzed (b) *Teff* straw.

Figures 1(a) and 1(b) show the morphological features of raw and acid hydrolyzed *Teff straw.*Figure 1(a) shows regular and compact surface structure with fibers arranged in bundles in *Teff* straw. The surface morphology of the acid hydrolyzed T*eff straw* changed as compared to raw *Teff straw*. Dilute sulfuric acid pretreatment could destroy the cellulose-hemicellulose-lignin structure, thereby removing some of the external fibers. Thus, the lignin and hemicellulose of the dilute H_2_SO_4_ acid pretreated *Teff* straw were partially removed and broken or became loose. The exposure of internal structures of *Teff* straw with acid increases the accessibility of cellulose for further processing [10]. The results of SEM analysis (Figure 1(b)) indicated that acid hydrolysis of T*eff* straw showed significant surface modification, i.e., developed honeycomb-like rough surfaces, nonuniform pores, and cavities caused by the reaction between H_2_SO_4_ and ester bonds, which led to removal of lignin and hemicellulose with cellulose domination [7].

### 3.4. XRD Analysis of Raw and Acid Hydrolyzed *Teff* Straw

**Figure 2:**
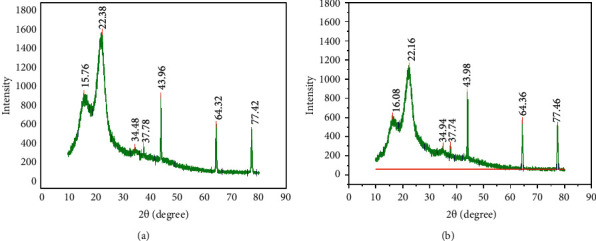
XRD analysis of raw (a) and acid hydrolyzed (b) *Teff* straw.

Figures 2(a) and 2(b) depict XRD patterns of raw and acid hydrolyzed *Teff* straw. The percentage of crystallinity of raw *Teff* straw was 64.12% whereas the percentage of crystallinity of acid hydrolyzed *Teff* straw was 65.7044%. showed that the pretreated rice straw by dilute acid has higher crystallinity degree (67.2%) when compared to native rice straw (59.37%), so the increased crystallinity index after pretreatment might be due to hydrolysis of glycosidic linkages in the cellulose accessible regions. The percentage of crystallinity of acid hydrolyzed *Teff* straw is higher than that of the raw *Teff* straw due to the removal of hemicellulose and amorphous parts of cellulose in the hydrolysate and due to hydrolysis of glycosidic linkages in the cellulose accessible regions, so the crystallinity of *Teff* straw is comparable with rice straw. In general terms, the acid hydrolyzed *Teff* straw generates better-defined signals than the raw one, which shows the elimination of noncrystalline components due to the effects of pretreatments, added to a slight increase in the signals that denote the crystalline structure of cellulose.

### 3.5. Effects of Dilute Acid Hydrolysis of *Teff* Straw

**Figure 3:**
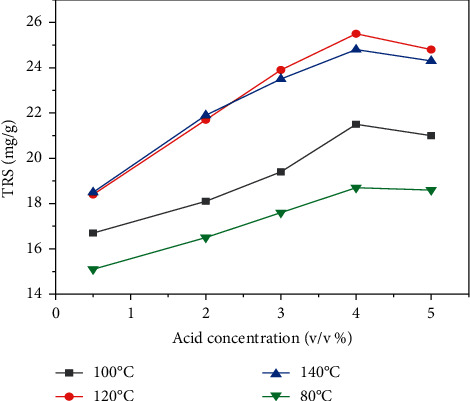
The effect of dilute sulfuric acid concentration and hydrolysis temperature on the yield of total reducing sugar (TRS) from *Teff* straw (hydrolysis time = 60 min and solid to liquid ratio = 1 : 20).

Figure 3 depicts the effect of dilute sulfuric acid and hydrolysis temperature at hydrolysis time of 60 min and solid to liquid ratio of 1 : 20 on the yield of total reducing sugar (TRS) from *Teff* straw. As shown in Figure 3, for all dilute acid concentrations, the yield of total reducing sugar (TRS) increased as the hydrolysis temperature increased from 80° to 140°C. This is due to the fact that higher temperature leads to more hydrolysis of the hemicellulose and amorphous portion of the cellulose. For hydrolysis temperatures of 100°, 120°, and 140°C, the yield of total reducing sugar (TRS) from *Teff* straw decreased as the acid concentration exceeded 4 v/v (%). This might be due to the degradation of monomeric sugars in the total reducing sugar to their degradation products especially furfural and 5-hydroxymethyl furfural (5-HMF). The maximum total reducing sugar (TRS) yield of 25.5 mg/g was obtained at a temperature of 120°C and acid concentration of 4 v/v (%), and the minimum total reducing sugar (TRS) yield of 15.1 mg/g was obtained at a hydrolysis temperature of 80 and acid concentration of 0.5 v/v (%). The minimum total reducing sugar (TRS) was obtained at lower temperature and acid load. This is because at lower temperature and acid concentration, it is hard to extract total reducing sugar (TRS) from the lignocellulosic biomass.

**Figure 4:**
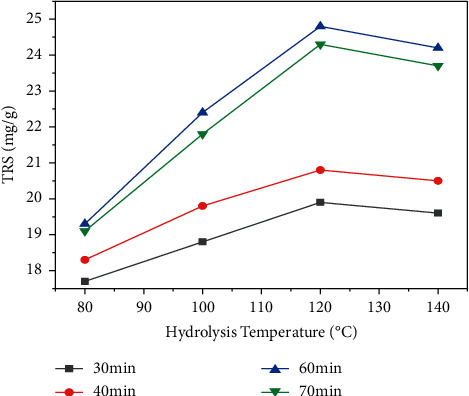
The effect of hydrolysis temperature and hydrolysis time on the yield of total reducing sugar (TRS) from *Teff* straw (dilute sulfuric acid concentration = 4 v/v (%) and solid to liquid ratio = 1 : 20).

Figure 4 shows the effect of temperature and hydrolysis time on yield of total reducing sugar (TRS) from *Teff* straw at dilute sulfuric acid concentration of 4 v/v (%) and solid to liquid ratio of 1 : 20. As depicted in the Figure 4, the yield of total reducing sugar (TRS) decreased as the hydrolysis temperature exceeded 120°C for all values of hydrolysis time. The maximum total reducing sugar (TRS) yield of 24.1 and 24.8 mg/g was obtained at 120°C for 60 min and 70 min, respectively. These results showed that hydrolysis temperature of 120°C and longer hydrolysis time favor the extraction of hemicellulose and amorphous portion of cellulose. However, for hydrolysis temperatures beyond 120°C and longer time, there might be decomposition of monomeric sugars to their degradation products. For lower temperature (80°C), the yield of total reducing sugar (TRS) from *Teff* straw was minimum for the shorter hydrolysis time (30 min and 40 min) considered (Figure 4). This is because at lower temperature and shorter hydrolysis time, extraction of total reducing sugar (TRS) from *Teff* straw was difficult.

**Figure 5:**
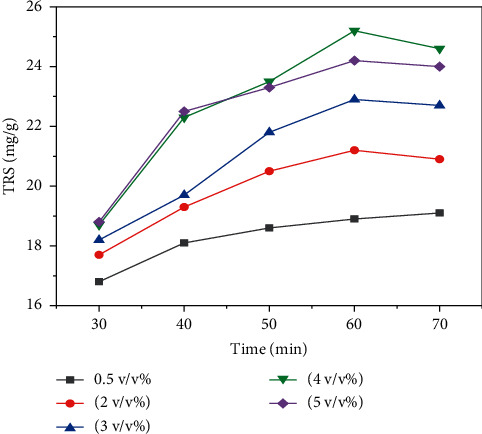
The effect of hydrolysis time and dilute sulfuric acid concentration on the yield of total reducing sugar (TRS) from *Teff* straw (hydrolysis temperature = 120°C and solid to liquid ratio = 1 : 20).

Figure 5 depicts the effect of hydrolysis time and dilute sulfuric acid concentration on yield of total reducing sugar (TRS) from *Teff* straw (hydrolysis temperature = 120°C and solid to liquid ratio = 1 : 20). Figure 5 depicts the maximum total reducing sugar (TRS) yield of 21.2 mg/g, 22.9 mg/g, 24.2 mg/g, and 25.2 mg/g for dilute sulfuric acid concentration of 2 v/v, 3 v/v, 5 v/v, and 4 v/v (%), respectively, from *Teff* straw at hydrolysis time of 60 min. The more total reducing sugar (TRS) yield for 4 v/v (%) sulfuric acid concentration than 5 v/v (%) sulfuric acid concentration at 60 min hydrolysis time is essentially due to the degradation of monomeric sugars to their degradation products at higher acid concentration and longer hydrolysis time. The yield of total reducing sugar (TRS) from *Teff* straw was minimal for all sulfuric acid concentrations considered in the experiments for shorter hydrolysis time (Figure 5).

**Figure 6:**
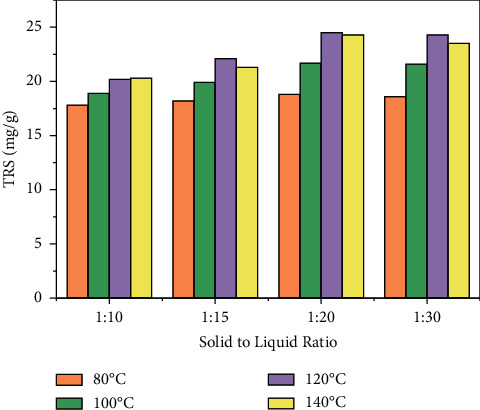
The effect of solid to liquid ratio and hydrolysis temperature on the yield of total reducing sugar (TRS) from *Teff* straw (dilute sulfuric acid concentration = 4 v/v (%) and hydrolysis time = 60 min).

Figure 6 shows the effect of solid to liquid ratio and hydrolysis temperature on the yield of total reducing sugar (TRS) from *Teff* straw (dilute sulfuric acid concentration = 4 v/v (%) and hydrolysis time = 60 min). Figure 6 shows slight variation in the yield of total reducing sugar (TRS) from *Teff* straw for specific hydrolysis temperatures (e.g., at 80°C) as the solid to liquid ratio varied from 1 : 10 to 1 : 30. The maximum total reducing sugar (TRS) yield of 24.5 mg/g was obtained at a solid to liquid ratio of 1 : 20 and hydrolysis temperature of 120°C from *Teff* straw. Figure 6 indicates that the total reducing sugar (TRS) yield (mg/g) increased as the solid to liquid ratio varied from 1 : 10 to 1 : 30. This was due to excess of liquid phase in hydrolysis mixture (solid to liquid ratio of 1 : 20 and 1 : 30) which helps in maximum hydrolysis of hemicellulosic and amorphous fraction of cellulose. Large excess of liquid phase also reduces bulk concentration of monomeric sugars resulting from hydrolysis, which helps in minimizing their further degradation to furfural and 5-HMF in the reaction mixture.

### 3.6. Optimization of Dilute Sulfuric Acid Hydrolysis on TRS Yield from *Teff* Straw

The second order polynomial model equation for the fitted data using coded values of independent variables and total reducing sugar (TRS) yield as a response variable is as follows:

(13) $$\begin{array}{c} \text{Total reducing sugar}\left(\text{TRS},\text{mg}/\text{g}\right)=+6.02-1.44A+0.07B-0.006C+0.34\;D\\ +0.03A^{2}-1.04\times 10^{-4}B^{2}+4.81\times 10^{-4}C^{2}-4.17\times 10^{-5}D^{2}+0.03AB+0.02AC\\ -0.03AD+1.00\times 10^{-3}BC-1.75\times 10^{-3}-1.83\times 10^{-3}CD. \end{array}$$

Table 1 depicts the experimental results for the dilute acid hydrolysis of *Teff* straw for total reducing sugar (TRS) yield. The maximum total reducing sugar of 26.3 mg/g was obtained at an experimental run number of 20, temperature of 120°C, acid concentration of 4% v/v, hydrolysis time of 45 minutes, and solid to liquid ratio of 1 : 20. On the other hand, the minimum yield of 15.9 mg/g was obtained at an experimental run number of 14, temperature of 80°C, acid concentration of 0.5 v/v %, solid to liquid ratio of 1 : 20, and hydrolysis time of 45 minutes. The results of maximum and minimum yield of total reducing sugar (TRS) reveal that at lower temperature and acid concentration, the total reducing sugar (TRS) extraction was also lower. At higher temperature, the increase in the total reducing sugar (TRS) yield might be due to sufficient temperature and acid concentration to hydrolyzed *Teff* straw whereas the lower temperature was not sufficient enough to hydrolyzed *Teff* straw.

Analysis of variance (ANOVA) for the fitted quadratic model is shown in Table 5. The model summary for regression coefficients (R^2^ = 99.6%, adjusted R^2^ = 99.1%, and predicted R^2^ = 97.5%) shows that the quadratic model fits into the experimental data. ANOVA study for the quadratic model was used to evaluate the impact and significance of terms in the regression equation. From the ANOVA results shown in Table 5, the *p* values for all the linear and interaction coefficients are <0.05, which shows that all variables and their interaction have significant effect on dilute sulfuric acid hydrolysis of *Teff* straw. The Lack of Fit with *F* value and *p* value of 8.29 and 0.11, respectively, indicates that Lack of Fit is not significant as compared to the pure error or, in other words, the model was significant.

DF is the degree of freedom; SS is the sum of squares; MS is the mean square; *p* values are significant at *p* ≤ 0.05; R^2^ = 0.996; predicted R^2^ = 0.975; adjusted R^2^ = 0.991.

**Figure 7:**
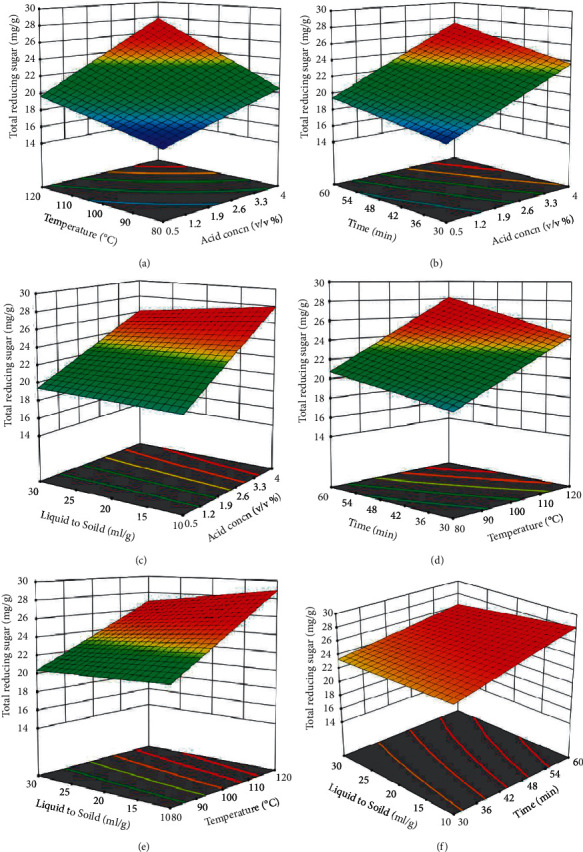
Surface plots showing the effect of (a) temperature and dilute sulfuric acid concentration, (b) dilute sulfuric acid concentration and hydrolysis time, (c) dilute sulfuric acid concentration and liquid to solid ratio, (d) temperature and hydrolysis time, (e) temperature and liquid to solid ratio, and (f) liquid to solid ratio and hydrolysis time on the total reducing sugar yield.

The 3D surface plots (shown in Figure 7), which are graphical representation of regression model equation (13), represent infinitive number of combinations of two test variables, with the third and fourth variables maintained at zero (center point) level. The contours were plotted to observe the interaction of two independent variables. An elliptical and elliptical nature contour plot are obtained by the interaction of temperature and dilute sulfuric acid concentration (Figure 7(a)), dilute sulfuric acid concentration and hydrolysis time (Figure 7(b)), dilute sulfuric acid concentration and liquid to solid ratio (Figure 7(c)), temperature and hydrolysis time (Figure 7(d)), temperature and liquid to solid ratio (Figure 7(e)), and liquid to solid ratio and hydrolysis time (Figure 7(f)) on the total reducing sugar yield depicting significant interaction between the variables. This is also confirmed by the *F* and *p* values of their interaction coefficients in the ANOVA analysis.

The optimum conditions for dilute sulfuric acid hydrolysis of *Teff* straw for maximum total reducing sugar yield of 26.65 mg/g under this model were as follows: concentration of H_2_SO_4_ = 4% v/v, temperature = 120°C, solid to liquid ratio = 1 : 20, and hydrolysis time = 55 min.

### 3.7. Validation of Experiments

Optimum conditions for maximum total reducing sugar yield predicted by the BBD experiments and RSM analysis have been corroborated by validation experiments of dilute sulfuric acid hydrolysis of *Teff* straw. The validation experiments were conducted in triplicate under optimized conditions (H_2_SO_4_ concentration = 4% v/v, temperature = 120°C, hydrolysis time = 55 min, and solid to liquid ratio = 1 : 20 g/ml) to ascertain reproducibility of results. The results of validation experiment under the optimum conditions agreed well with model predictions.

## 4. Conclusion

The morphological analysis using SEM showed that hydrolyzed *Teff* straw with dilute sulfuric acid has more pores and distorted bundles than those of raw *Teff* straw. XRD analysis also showed that hydrolyzed *Teff* straw has higher crystallinity and smaller crystallite size than raw *Teff* straw, which might be due to removal of hemicellulose, amorphous cellulose, and lignin components. Under the optimized conditions for dilute sulfuric acid hydrolysis of *Teff* straw (120°C, 4% v/v H_2_SO_4_ concentration, 1 : 20 solid to liquid ratio, and 55 min hydrolysis time), we have found a total reducing sugar yield of 26.65 mg/g. The results of the present study showed that *Teff* straw is good potential lignocellulosic biomass for the production of biofuels and value-added chemicals.

## Figures and Tables

**Table 1:** Coded and actual value levels of the variables for Box–Behnken design.

Independent variables	Symbol	Coded and actual value levels		
		−1	0	+1
Acid concentration (% v/v)	A	0.5	2.25	4
Hydrolysis temperature (^o^C)	B	80	100	120
Hydrolysis time (min)	C	30	45	60
Liquid to solid ratio (ml/g)	D	10	20	30

**Table 2:** Experimental results for the dilute acid hydrolysis of *Teff* straw for total reducing sugar (TRS) yield.

Run No	Acid concentration (v/v %)	Temperature (^o^C)	Hydrolysis time (min)	Liquid to solid ratio (ml/g)	TRS (mg/g)
1	2.25	80.00	30.00	20.00	16.7
2	4.00	100.00	45.00	10.00	23.5
3	2.25	80.00	45.00	30.00	18.1
4	2.25	100.00	45.00	20.00	20.1
5	2.25	120.00	60.00	20.00	24.3
6	0.50	100.00	45.00	10.00	16.4
7	2.25	80.00	60.00	20.00	19
8	2.25	120.00	45.00	10.00	22.8
9	2.25	100.00	45.00	20.00	20.1
10	0.50	100.00	45.00	30.00	17.9
11	2.25	100.00	60.00	10.00	21.5
12	2.25	100.00	60.00	30.00	21.2
13	2.25	100.00	30.00	30.00	19.5
14	0.50	80.00	45.00	20.00	15.9
15	2.25	100.00	45.00	20.00	20.1
16	2.25	120.00	45.00	30.00	22.6
17	2.25	100.00	30.00	10.00	18.7
18	0.50	120.00	45.00	20.00	18.7
19	2.25	80.00	45.00	10.00	16.8
20	4.00	120.00	45.00	20.00	26.3
21	2.25	120.00	30.00	20.00	20.8
22	4.00	100.00	30.00	20.00	21.4
23	0.50	100.00	60.00	20.00	18.5
24	4.00	100.00	60.00	20.00	24.5
25	0.50	100.00	30.00	20.00	16.9
26	4.00	80.00	45.00	20.00	19.6
27	4.00	100.00	45.00	30.00	23.1

**Table 3:** Results of proximate analysis of *Teff* straw as compared to other biomass sources.

Biomass sources	Moisture (%)	Volatile matter (%)	Ash content (%)	Fixed carbon (%)	References
*Teff straw*	6.7	78.5	12.5	2.3	*Present study*
Rice hulls	9.0	70.8	11.1	18.1	[21]
Wheat straw	7.9	76.9	4.0	19.1	[22]
Sugarcane bagasse	4.5	77.1	2.4	16.0	[23]
Rice straw	6.9	58.6	20.0	14.8	[24]
Switch grass	9.1	73.1	3.8	23.0	[25]
Corn cob	11.7	72.3	10.7	4.9	[24]
Water hyacinth	2.9	67.1	13.9	18.9	[26]

**Table 4:** Results for chemical composition analysis of *Teff* straw compared with other biomass sources.

Biomass	Extractive (wt%)	Cellulose (wt%)	Hemicellulose (wt%)	Lignin (wt%)	References
*Teff straw*	3.2	41.8	38	17	*Present study*
Wheat straw	3	38	37	17	[22]
Sugarcane bagasse	3.37	40.84	30.79	25	[8]
Rice straw	3.22	35	24.3	17.73	[27]
Switch grasses	—	45	31.4	12	[6]
Corn cob	—	41.27	46	7.40	[28]
Water hyacinth	—	31.67	27.33	3.93	[29]
Pineapple waste	11	30	37	22	[11]
Barely straw	—	14–19	27–38	31–45	[30]

**Table 5:** ANOVA for the quadratic model for dilute sulfuric acid hydrolysis of *Teff* straw.

Source	SS	DF	MS	*F* value	*p* value
Model	194.91	14	13.92	196.74	<0.0001
A	97.47	1	97.47	1377.40	<0.0001
B	71.54	1	71.54	1010.98	<0.0001
C	18.50	1	18.50	261.44	<0.0001
D	0.75	1	0.75	10.60	0.0069
AB	3.80	1	3.80	53.74	<0.0001
AC	0.64	1	0.64	9.04	0.0109
AD	0.90	1	0.90	12.75	0.0038
BC	0.36	1	0.36	5.09	0.0436
BD	0.49	1	0.49	6.92	0.0219
CD	0.30	1	0.30	4.97	0.0441
A^2^	0.049	1	0.049	0.69	0.4217
B^2^	9.259E-003	1	9.259E-003	0.13	0.7238
C^2^	0.063	1	0.063	0.88	0.3655
D^2^	9.259E-005	1	9.259E-005	1.308E-003	0.9717
Residual	0.85	12	0.071		
Lack of Fit	0.83	10	0.083	8.29	0.1123
Pure error	0.020	2	1.0E-002		
Corr total	195.76	26			

## Data Availability

The datasets generated and/or analyzed during the current study are available from the corresponding author on reasonable request.

## Abbreviations

ANOVA: Analysis of variance
AR: Analytical reagent
BBD: Box–Behnken design
CI: Crystalline index
DOE: Design of experiment
DSC: Differential scanning calorimetry
FC: Fixed carbon
Ff: Fehling factor
FTIR: Fourier transform infrared spectroscopy
LCM: Lignocellulose material
MC: Moisture content
RS: Reducing sugar
RSM: Response surface methodology
SEM: Scanning electron microscopy
TRS: Total reducing sugar
VM: Volatile matter
XRD: X-ray diffraction.
